# A Patient Presenting with Bilateral Lung Lesions, Pleural Effusion, and Proteinuria

**DOI:** 10.1155/2013/489362

**Published:** 2013-05-09

**Authors:** Katerina D. Samara, Giorgos Papadogiannis, Andrew G. Nicholson, Eleutherios Magkanas, Konstantinos Stylianou, Nikolaos Siafakas, Katerina M. Antoniou

**Affiliations:** ^1^Department of Thoracic Medicine, University of Crete, Medical School, 71110 Heraklion, Crete, Greece; ^2^Pathology Department, Royal Brompton Hospital, SW3 6NP London, UK; ^3^Radiology Department, University of Crete, Medical School, 71110 Heraklion, Crete, Greece; ^4^Nephrology Department, University of Crete, Medical School, 71110 Heraklion, Crete, Greece

## Abstract

Diagnosis and management of a systemic vasculitis are among the most demanding challenges in clinical medicine. A patient with a past history of cryptogenic organizing pneumonia presents with new bilateral lung lesions, unilateral pleural effusion, and significant proteinuria. The patient tested p-ANCA and anti-MPO positive but c-ANCA negative. A diagnosis of granulomatosis with polyangiitis GPA was reached after performing both renal and lung biopsies. Step-by-step differential diagnosis and management are discussed.

A 71-year-old male patient was referred to the department of thoracic medicine from the nephrology department for further investigation of an abnormal chest CT. The patient, a lifelong nonsmoker, working as a farmer, had initially presented to the emergency department two months ago complaining of malaise, pleuritic chest pain, and dry cough. He was under treatment for arterial hypertension (amlodipine and irbesartan) and had a medical history of benign intestinal polyposis and a diagnosis of cryptogenic organizing pneumonia (COP) five years ago, histologically proven through lung biopsy. His family history is unremarkable. Upon initial evaluation, he was admitted to the nephrology department due to elevated creatinine serum levels and significant proteinuria on a random urine specimen.

On admission, the patient was afebrile with normal vital signs (RR: 15/min, BP: 135/80 mmHg, HR: 87 bpm, SatO_2_: 96% on room air). Chest physical examination revealed bilateral expiratory wheezing in both lungs. Physical examination of the cardiovascular system and the abdomen was unremarkable. The chest radiograph revealed increased opacity in the left lower pulmonary field associated with absence of the same side costophrenic angle (Figure 1). A renal ultrasound was normal. Laboratory findings revealed elevated WBC, normal hematocrit, and platelet count. Serum renal markers (BUN and creatinine) were elevated as was ESR (85 mm/1 h). A random urine specimen examination revealed significant proteinuria (7034 mg) and urinary sediment analysis showed pus cells 5–10 per field, dysmorphic erythrocytes 5–10 per field, a few squamous epithelial cells, hyaline casts 2 per field, granular casts 3 per field, and mixed casts (leukocytic-epithelial) 3 per field. The examination of the urine for the existence of Bence-Jones protein was negative. Serum IgA, IgG, and IgM levels were normal. Thyroid hormone levels were normal. He underwent a renal biopsy, due to elevated levels of p-ANCA and anti-MPO (1 : 160, 1 : 300 respectively), while c-ANCA and anti-Pr3 levels were normal. The renal biopsy revealed membranous glomerulonephritis, with findings of regional necrotic lesions within glomeruli (Figures 2(d) and 2(f)).

Based on the renal biopsy findings, further investigation was initiated. The patient tested negative for HCV, HBV, and HIV. He denied any drug history compatible with the renal biopsy findings. Finally, a chest contrast enhanced CT was performed that revealed bilateral multiple nodular opacities in all lung fields with a left-sided mild pleural effusion. A mass-like lesion with hypoattenuated area was depicted in the left costophrenic angle (Figures 2(a) and 2(b)). The patient was then transferred to the department of thoracic medicine from the nephrology department for further investigation. 

Pulmonary function assessment was within normal limits except for diffusion capacity which was elevated. A thoracocentesis was performed and revealed a lymphocytic exudative pleural effusion, with normal pH (glucose: 103 mg/dL, total protein: 3,6 g/dL, LDH: 365 U/lt, alb: 1,9 md/dL). Culture of the fluid was sterile, and the cytological examination negative for malignancy. Bronchoscopy revealed no evidence of endobronchial obstruction or submucosal infiltration. The BAL was not bloody, the BAL culture was sterile, and the cytological examination was negative for malignancy. A CT-guided lung biopsy was performed. The biopsy showed some features of organizing pneumonia such as intra-alveolar organization associated with a nonspecific chronic inflammatory cell infiltrate. In addition, there were areas of basophilic necrosis containing neutrophilic debris, the latter centered on the pulmonary vasculature, with focal fragmentation of the elastin layers. Occasional histiocytic giant cells were noted, although no discrete granuloma was seen. ZN and Grocott staining for organisms were negative (Figure 2(e)). The features were classified, by an expert histopathologist, as a pulmonary vasculitis, morphologically closest to granulomatosis with polyangiitis (GPA). 

Granulomatosis with polyangiitis (GPA) is a rare multisystem autoimmune disease of unknown etiology [1, 2]. Its hallmark features include necrotizing granulomatous inflammation and pauci-immune vasculitis in small- and medium-sized blood.

In the case reported here, three mainly clinical features should be highlighted: (i) the presence of membranous glomerulonephritis, (ii) p-ANCA and anti-MPO positivity with pleural effusion, and finally (iii) histologically confirmed pulmonary vasculitis associated with features compatible with OP histology.

Primarily, the presence of membranous glomerulonephritis should always initiate a thorough work-up to exclude the possibility of malignancy. It is not uncommon for adults over the age of 60 years to have an underlying carcinoma (especially lung, colon, stomach, or breast). Our patient underwent a thorough investigation, with repeated cytological examination of BAL and pleural fluid and finally lung biopsy to ensure that the possibility of lung cancer is ruled out [3, 4]. Secondly, p-ANCA and anti-MPO positivity with normal levels of c-ANCA and anti-PR3 do not commonly correlate with the diagnosis of GPA. Approximately 82%–94% of patients with either GPA or MPA are ANCA positive, depending on the severity of the disease. GPA is primarily associated with PR3-ANCA positivity, while MPA with anti-MPO positivity. The sensitivity of c-ANCA PR3 is 85%–90%, 60%, and 40% in generalized active GPA, limited pulmonary GPA, and GPA in remission, respectively. Our patient was anti-MPO positive, a finding that was inconsistent with the result of the lung biopsy. However, 20% of patients with GPA or MPA have the alternative ANCA type and at least 10% of patients are ANCA negative. 

Moreover, we underline the findings of our patient's chest CT [5–7]. The CT examination revealed multiple nodules in the lung parenchyma and one large mass-like lesion with hypo-attenuated area in the left costophrenic angle. The presence of mild pleural effusion was also depicted at the left side. The imaging spectrum of GPA is very wide and nonspecific [8, 9]. The most common radiological findings on chest CT are nodules and masses, usually multiple and bilateral, demonstrating subpleural or bronchovascular distribution. These lesions are typically diffused, and approximately 25% are cavitated [5–9]. Pleural involvement is not as common as parenchymal pulmonary manifestations (estimated incidence 10%) especially as the first manifestation of GPA [10–12]. Moreover, the pathophysiology of pleural effusion in immunologic diseases has to be further investigated. Our group has recently described a marked NLRP3 inflammasome activation with increased production of IL-1*β* suggesting a possible novel pathogenetic pathway [13]. The first priority was to exclude the presence of lung cancer through biopsy [3]. 

Finally, it should be reminded that our patient had a past diagnosis of cryptogenic organizing pneumonia (COP), histologically proven via thoracoscopic lung biopsy. Pathologists need to be aware that GPA can occasionally manifest histological changes suggestive of OP. It is not unlikely that our patient demonstrated this histological variant of GPA five years ago. However, in this case, the patient would be sicker at presentation.

 Travis et al. [14] described the histopathologic manifestations of pulmonary Wegener's granulomatosis, dichotomized as major and minor. Bronchiolitic obliterans as a minor manifestation was found in 31% of the specimens, while it is important to highlight that minor lesions represent the dominant pattern in about 20% of cases [14, 15]. Katzenstein and Locke studied the histological manifestations of 25 patients with GPA and solitary lesions and reported three cases that had prominent features of bronchiolitis obliterans-organizing pneumonia [16]. Moreover, more relevant to the current case, 16 patients with the BOOP-like variant of GPA were described [17].

One more point that we have to discuss is that the histology was based on core biopsy and thus could explain why all the characteristics findings were not fully present. However, our expert pathologist was able to give a definitive diagnosis, always aware of the clinico-radiologica data of this patient. It is of note that safety and diagnostic accuracy of image-guided core biopsy of thoracic lesions make it a useful tool in the assessment of disease activity in WG patients with persistent chest radiographic lesions [18]. On the other hand, CT-guided FNB has lower diagnostic accuracy and higher complication rate than those of larger pulmonary lesions in the diagnosis and management of small pulmonary nodules (<10 mm) [19]. 

It is extremely important to emphasize the significance of follow up for this patient. The patient was treated with pulse corticosteroids and IV cyclophosphamide. Six months later a follow-up chest CT was performed and revealed improvement of the imaging findings. The parenchymal nodules and the left-sided pleural effusion disappeared. The mass lesion at the left lung base diminished and the CT scan at this level showed the presence of a nodule. 

In conclusion, the diagnosis and management of a systemic vasculitis are among the most demanding challenges in clinical medicine.

## Figures and Tables

**Figure 1:**
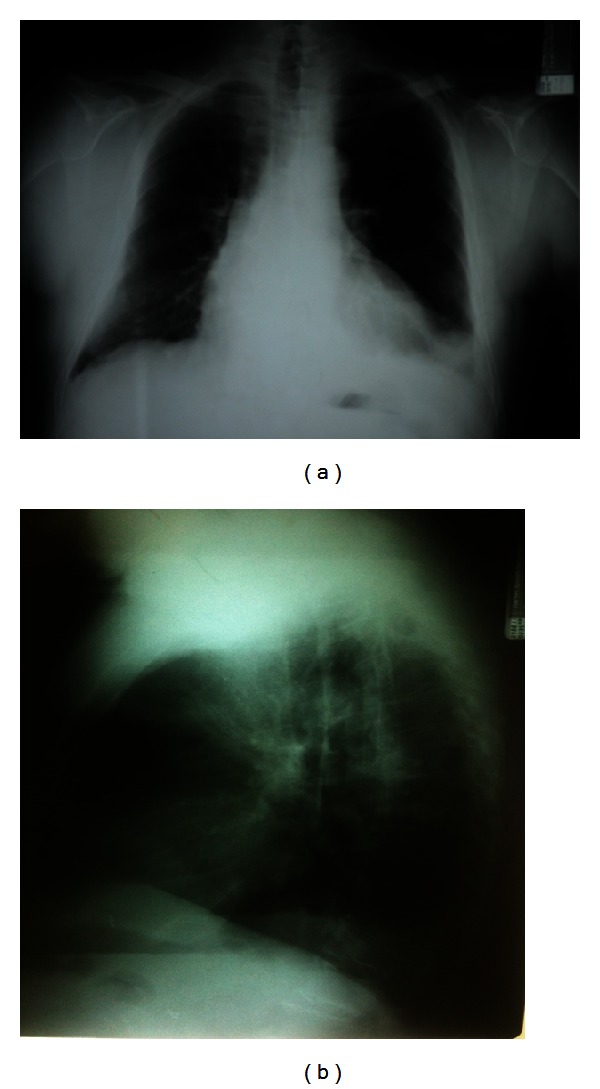
Chest X-ray of the patient on admission in the nephrology department.

**Figure 2:**
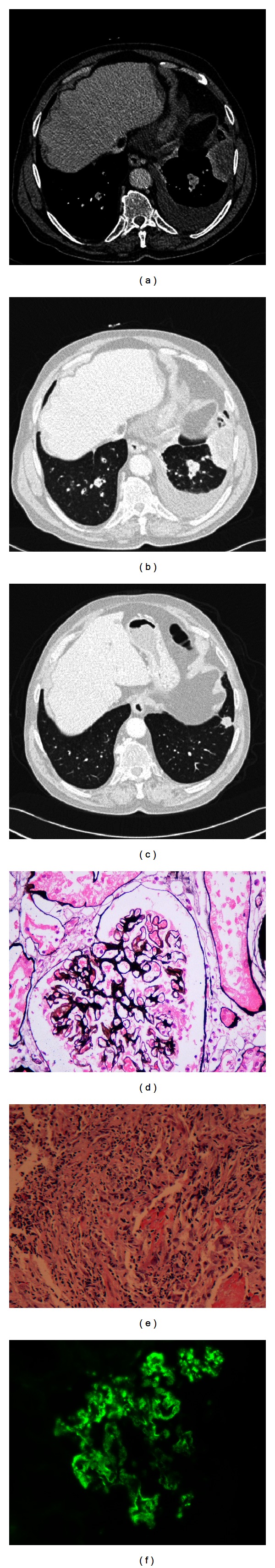
(a) Initial contrast enhanced CT reveals a mass-like lesion with hypoattenuated area in the left costophrenic angle. Pleural effusion is also noted in the left side. (b) Initial CT. Lung window scan reveals the mass-like lesion in the left costophrenic angle. Nodular lesions are also visible bilaterally. (c) Follow-up CT. Lung window settings, at the same level eight months later, show a nodule in the left costophrenic angle. The parenchymal nodules have disappeared. (d) Mild, diffuse, nonhomogeneous thickening of capillary basal membrane with rare micro-vacuolar degeneration of the basal membrane (Silver, ×400). (e) CT-guided lung biopsy shows a core of alveolar parenchyma in which there is intra-alveolar organisation associated with a non-specific chronic inflammatory cell infiltrate. In addition, there are areas of basophilic necrosis containing neutrophilic and fibrinoid debris. Occasional histiocytic giant cells are noted within the inflammatory cell infiltrate, although no definitive coalescent granuloma is seen. EVG staining shows that this neutrophilic/necrotic infiltrate is focally centred on the walls of the pulmonary vasculature with some loss and fragmentation of the elastin layers. (f) Non-homogeneous of IgG immunoglobulin along the capillary basal membrane walls exhibiting granular or pseudolinear distribution (IF, ×400).
